# ADNP prompts the cisplatin-resistance of bladder cancer via TGF-β-mediated epithelial-mesenchymal transition (EMT) pathway

**DOI:** 10.7150/jca.58049

**Published:** 2021-06-22

**Authors:** Yu Xie, Shuai Zhu, Jinglei Zang, Guanlin Wu, Yuheng Wen, Yu Liang, Ying Long, Weiming Guo, Chuanbing Zang, Xiang Hu, Gang Fan, Shuanglin Xiang, Jian Zhang

**Affiliations:** 1 Key Laboratory of Protein Chemistry and Developmental Biology of the Ministry of Education, Hunan Normal University, 410081 Changsha, China.; 2 Department of Urology, the Affiliated Cancer Hospital of Xiangya School of Medicine, Central South University, Hunan Cancer Hospital, 410013 Changsha, China.; 3 Changsha Health Vocational College, 410600 Changsha, China.; 4 Department of Pathology, School of Basic Medical Sciences, Fudan University, 200433 Shanghai, China.; 5 Pingxiang Maternal and Child Care Hospital, 337000 Pingxiang, China.; 6 Clinical Translational Research Center, the Affiliated Cancer Hospital of Xiangya School of Medicine, Central South University, Hunan Cancer Hospital, 410013 Changsha, China.; 7 The 2nd Affiliated Hospital of South China University, 421001 Hengyang, China.; 8 Medizinische Klinik m. S. Hämatologie u. Onkologie, Campus Bejamin Franklin, Unviersitätsmedizin Berlin Charité, 12203 Berlin, Germany.; 9 Department of Urology, Huazhong University of Science and Technology Union Shenzhen Hospital; the 6th Affiliated Hospital of Shenzhen University Health Science Center, 518060 Shenzhen, China.

**Keywords:** ADNP, bladder cancer, epithelial-mesenchymal transition, chemoresistance, TGF-β/Smad

## Abstract

Activity-dependent neuroprotective protein (ADNP) is vital for embryonic development and brain formation. Besides, the upregulated expression of ADNP enhances tumorigenesis in some human tumors like bladder cancer (BC). However, the potential roles of ADNP in drug resistance and the related mechanisms in BC is unknown. We performed this study to elucidate the influence of ADNP in the chemoresistance of BC and tried to explore the underlying molecular mechanism. The expressions of ADNP in BC from progression and non-progression patient specimens were measured by quantitative real-time PCR (qRT-PCR) and immunohistochemistry (IHC). *In vitro* experiments including colony formation, cell counting kit-8 (CCK-8), wound healing, and *in vivo* tumorigenesis assay were performed to explore the effects of ADNP on chemoresistance of BC. The impacts of ADNP on TGF-β/Smad signaling pathways were explored by western blot. Our results showed that the expression of ADNP mRNA and protein were significantly upregulated in BC tissues of the patients who suffered tumor-progression via RT-PCR and western blot. Cox regression survival analysis revealed that patients with high ADNP expression closely linked to shorter tumor-free survival. ADNP downregulation in BC showed more sensitive to cisplatin *in vivo*, while ADNP overexpression showed the opposite results. Additionally, we confirmed that ADNP promoted cell migration and EMT, thereby inducing cisplatin resistance, which may be related to TGF-β / Smad signaling pathway.

## Introduction

Bladder cancer (BC) is one of the most common malignancies of the human urinary system 1. Chemotherapy remains a vital therapeutic strategy for BC 2, 3. Despite significant advances in BC research in the past few decades, the survival of patients with BC still faced severe challenges 4. So there is urgent need to explore the underlying mechanisms of chemoresistance in BC. Previous studies indicated a molecular and phenotypic association between chemoresistance and the acquisition of the epithelial-mesenchymal transition (EMT)-like phenotype in many types of cancers such as breast cancer, glioblastoma, hepatocellular carcinoma 5-9. For example, EMT involved in chemoresistance of cancer cells against conventional therapeutics including cisplatin, vincristine and oxaliplatin 10. On the mechanism, TGFβ signaling was associated with chemoresistance and EMT characteristics, since TGFβ-induced EMT increased chemoresistance in hepatocellular carcinoma.

Activity-dependent neuroprotective protein (ADNP) is a protein that plays a crucial role in neuroprotective responses to cellular growth and proliferation 11. ADNP was frequently amplified and overexpressed in many human malignancies, such as ovarian 12. ADNP second cDNA clone (H3) differed from clone H7 in several polymorphic regions and polyadenylation sites. In addition, clone H3 contains frameshift mutations with premature termination codon at position 3408 13. The discovery of ADNP mutations in neurological disorders ignited an interest to explore the role of ADNP in cancer. Studies have shown that under different stress conditions, ADNP may mutate or differ in expression, thus accelerating the progression of cancer 14. Wild type ADNP can impact cellular proliferation via the WNT signaling pathway in colon carcinoma 15. However, the activation of ADNP signaling system, mediated by an endogenous pituitary adenylate cyclase-activating polypeptide, can increase the resistance of malignant peripheral nerve sheath tumor to H_2_O_2_-induced death with serum starvation 16. Our previous study has shown that overexpression of ADNP in BC stimulated the Akt-MDM2-p53 pathway and enhanced binding of cyclin D1 to CDK4 or CDK6, accelerating the cell cycle transition from G1 phase to S phase 17. ADNP may act as an oncogene in certain cellular contexts. Hence, we speculate that ADNP contribute to chemoresistance of BC through modulating EMT processes. In this study, we preliminarily investigated the relationship between chemoresistance of ADNP and BC, and the effect of ADNP on TGF-β signaling pathway of EMT.

## Materials and Methods

### Tissue samples and cell lines

All patients had non-muscle invasive bladder migration. The clinical stages included CIS, Ta and T1. Before inclusion, all papillary tumors were completely removed. Patients with T1 disease underwent urethrectomy. The included patients had sufficient bone marrow function (more han 1500 blood granulocytes/mm^3^ and more than 150,000 platelets/mm^3^). Patients were > 18 years old and had the ability to provide informed consent. Immunosuppressed patients (for example, use of HIV, chronic steroids) were excluded.

All of paraffin-embedded specimens of human non-muscle invasive bladder transitional cell carcinoma tissues were obtained from the patients who were recruited in the Affiliated Cancer Hospital of Xiangya Medical College of Central South University from January 2015 to December 2017. Another 43 non-muscle invasive bladder urothelial carcinoma surgical fresh specimens were also collected randomly, including 25 patients with progression BC and 18 patients with non-progression BC. All patients were diagnosed by histopathology and were treated with bladder tumor resection and intravesical chemotherapy. We defined non-progression BC by using urethrocystoscopy, CT, and urinary shedding, indicating no bladder tumor progression during 3-year follow-up. By 3-year follow-up biopsy, a mature invasive transitional cell carcinoma of the lamina propria is defined as progression BC.

Human BC cell lines, including T24, BIU87, 5637, and TCCSUP, were obtained from Sun Yat-sen University Cancer Center (Guangzhou), and cultured in RPMI-1640 medium (Gibco, USA) and 10% fetal bovine serum (Gibco, USA) + 1% Streptomycin and placed in a 5% CO2, 37 °C. Prior to the use of these clinical data for research, informed patient consent and approval from the institutional research ethics committee were obtained.

The study was approved by the ethics committee of the Affiliated Cancer Hospital of Xiangya School of Medicine, Central South University (No: 2016-0008). This study was conducted in accordance with the following ethical guidelines: the Helsinki Declaration, the International Code of Ethics in Biomedical Research (CIOMS) involving human subjects, the Belmont Report, and the American Common Rules.

### Quantitative real-time PCR (qRT-PCR)

mRNA were measured as previously described 18-20. Total RNA was extracted from the tissues using TRIzol reagent (Invitrogen, USA) according to the manufacturer's instructions. Spectrophotometry (A260 / A280 = 1.8-2.0) was used to determine RNA purity, and cDNAs were generated with M-MLV transcriptase according to the manufacturer's instructions (BioRAD, USA). The quantitative real-time PCR mixture system is 1 μg cDNA, 0.4 ul of the target gene primer, and 5 ul 2 × SYBR Green (BioRAD, USA). The reaction was performed using the LightCycler480 Real-Time PCR System (BioRAD, USA). The reaction procedure is: 1 min at 95 °C, followed by 35 cycles of 95 °C for 15 seconds, 95 °C for 10 seconds, 65 °C for 60 seconds, and finally 97 °C for 1 minute. The 2^-ΔΔCT^ method was used to determine the relative gene expression level of non-progression BC tissue and progression BC tissue. ADNP amplification primer sequences are:

- Forward: 5'-CATCCTGCGTCTGGACCTGG-3';
- Reverse: 5'-TAATGTCACGCACGATTTCC-3'.

### Western blot analysis

Western blot analysis was performed as previously described 21, tissues and cells were washed with PBS, and lysed with RIPA lysis buffer including protease lysis inhibitor (Roche USA) and phosphatase inhibitor (Roche USA). A BCA protein assay kit (Thermo Scientific, USA) was used to measure protein concentration. 30ug protein extract was separated with a 10% SDS-polyacrylamide gel and transferred to a PVDF membrane (microwell). The membrane was blocked with TBST buffer containing 5% skim milk at 37 °C for 2 hours, incubated with primary antibody at 4 °C overnight, and incubated with peroxidase-conjugated secondary antibody for 2 hours. The signal was detected using ECL kit (Cell Signaling Technology, 12757) and photographed. Bands were quantified using gray-scale analysis software (Bio-Rad). GAPDH expression is used as a loading control to standardize the expression of other proteins. The main antibodies are as follows: anti-ADNP (1: 1000, Proteintech, USA), anti-GAPDH, N-Cadherin, Vimentin, Snail, E-Cadherin, β- Catenin, Claudin-1, TGF-β, TGF-βR1, Smad2/3, p-Smad2/3 (1: 1000, Cell Signaling Technology, USA). The secondary antibodies are as follows: HRP-goat anti-rabbit Ig G, HRP-goat anti-mouse Ig G (1: 5000, Cell Signaling Technology, USA).

### Immunostaining

Immunostaining was performed as previously described 22, 23, the paraffin-embedded tissue was cut into 5 mm slices, and baked at 60 °C for 2 h. The slices dewaxed in xylene and hydrated in gradient ethanol. Endogenous enzymes were removed in 0.3% hydrogen peroxide. The slices were added into EDTA antigen repair buffer and heated. The first antibody was added for incubation (ADNP, E-Cadherin, N- Cadherin and Vimentin (1: 1000, Abcam ab199120) at 4 °C overnight. Slices were washed by PBS. Slices were incubated with HRP-conjugated goat -rabbit or goat-mouse secondary antibody at 37 °C. DAB is applied to color rendering. After the slices were dehydrated and transparent, they were sealed with neutral gum.

The immunostaining degree of paraffin embedded sections was scored independently by two experienced pathologists. Both of them were blind to the histopathological characteristics and clinical data of the specimens. All fields of a slice were analyzed. The score was based on the proportion and intensity of positive staining of tumor cells. The percentage score of tumor cells was as follows: 0: no positive tumor cells; 1: <10% tumor cell positive; 2: 10-35% positive tumor cells; 3: 36-75% positive tumor cells); 4.> 75% positive tumor cells. The staining intensity was graded according to the following standards: 1.no staining; 2. Weak staining (light yellow); 3. Moderate staining (light brown); 4. Strong staining (brown). The staining index is calculated as the product of the proportion of positive tumor cells and the staining intensity score. The staining index (0, 1, 2, 3, 4, 6, 8, 9, 12) was used to evaluate the expression of ADNP in BC. Staining index score ≥6 was defined as high expression, while score <6 was defined as low expression 24.

### Cell transfection

ADNP overexpression plasmid (ViGene, Shandong) and empty vector (ViGene, Shandong, China) were used. The ADNP expression construct was generated by cloning the full-length human ADNP cDNA into a pMSCV-retro-puro vector (Clontech, Palo Alto, CA). For ADNP knockdown, the trispecific knockdown site ADNP-shRNA (KD-1: CAACATGACTGATGGAGTA; KD-2: GCAAATGCCTCTACTGTAA; KD-3: TAGTAAGACTGCTGACAAA) and negative control shRNA (NC) were purchased from Shanghai Jikai, China Biotechnology. Cells were transfected with lentivirus when cells reached 30-50% confluency. Transfected cells were screened with 1 ug/ml puromycin (Sigma-Aldrich) after 72 h. The stable transfected cell line was maintained in a culture medium containing puromycin at 0.1 ug/ml.

### Cell viability assay

Cell viability were measured as previously described 25, 26. Approximately 4000 stably transfected cells were seeded on 96-well plates. The cells were treated with different concentrations of cisplatin for 24 h after adhered, 10 ul of Cell Counting Kit-8 reagent (CCK8, Dojindo Kumamoto, Japan) was added into each well and incubated for 4 h. The absorbance was measured with a spectrophotometer (Molecular Devices, Sunnyvale, CA, USA) at a wavelength of 450 nm.

### Colony growth assay

Approximately 3000 stably transfected cells were seeded on 6 well plates and cultured for 15 days. The medium was changed every 3 days. Cells were washed twice with PBS before being harvested, stained with crystal violet and photographed 27, 28. The stained colonies were counted with imageJ.

### Wound healing experiment

Stably transfected cells (1×10^6^) were cultured in 6 well plates until there was 60%-70% confluence. The scratch was made with a sterile pipette tip (10 μl), and the exfoliated cell was washed away with PBS. The images were obtained under the microscope and the wound distance was recorded and calculated with ImageJ at 0h and 24h, respectively. The migration percentage was calculated according to the following formula: Healing rate = [(0h distance - 24h distance)/0h distance] × 100% 29, 30.

### Tumor xenograft experiments

As previously described 31, 32, 4-week-old NOD/SCID nude mice were purchased from Hunan SJA Experimental Animal Company (Changsha, Hunan). Stable ADNP knockdown T24 cells and negative control T24 cells (1x10^6^ cells) were injected into the right armpit skin of nude mice (5 per group), and each nude mouse in the drug group were injected with 5 mg/kg of cisplatin once every two days from the day 7^th^ after injection. Control group were injected with equivalent PBS. The tumor length (L) and width (W) were measured with a vernier caliper every 5 days. The volume of the tumor was calculated according to the following formula: Volume= (L×W^2^)/2. Tumor weight was measured at the end of the experiment. Animals were sacrificed on the day 42nd. Tumors were isolated, weighed and immunostained with anti-ADNP, anti-N-cadherin, anti-E-cadherin and anti-Vimentin. The animal feeding and experimental operations were performed under SPF conditions and were approved by the Animal Ethics Committee of the Affiliated Cancer Hospital of Xiangya Medical College of Central South University. The study was approved by the animal ethics committee of the Affiliated Cancer Hospital of Xiangya School of Medicine, Central South University (No: 2019-007).

### Statistical analysis

All statistical analyses were performed using the SPSS 21.0 statistical software (SPSS Incorporated, Chicago). All data were from at least three independent repeated experiments. Mean ± SD significant tests were used, and the specific test was performed by the student test. Chi-square test was used to analyze the relationship between ADNP expression and clinicopathological characteristics. Survival curves were drawn using Kaplan-Meier method and compared by log-rank test. Survival data were analyzed using univariate and multivariate cox-regression. All cases were statistically significant with P <0.05.

## Results

### ADNP was up-regulated in patients suffered tumor-progression

**Figure 1:**
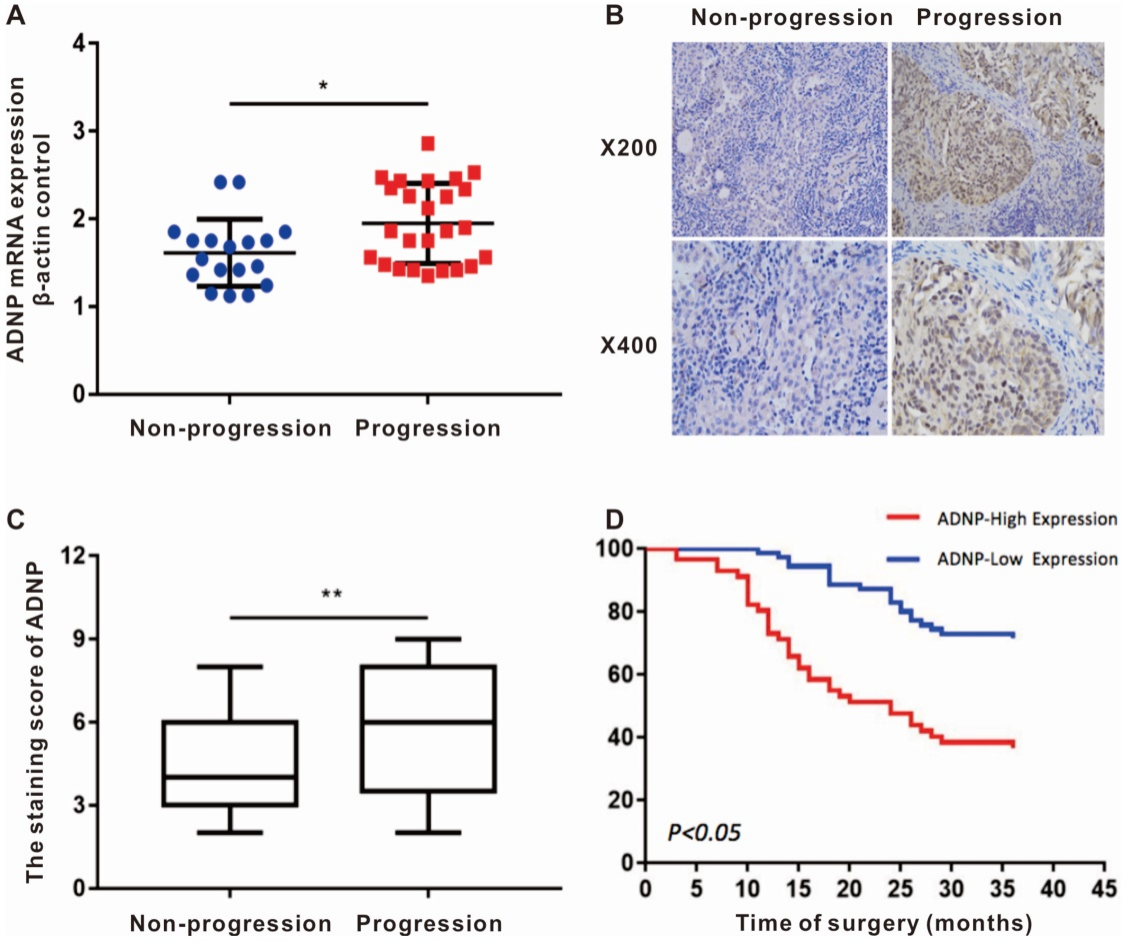
** ADNP expression associates with tumor progression in patients after intravesical chemotherapy. A,** ADNP mRNA in 43 bladder tumors, including 25 progression BC and 18 non-progression BC, were analyzed by using real-time quantitative PCR. Standardization was performed with β-actin as a control gene. **B,** Representative images of ADNP protein expression in non-progression BC and progression BC, the slides represent 50 microns. **C,** Quantitative analysis of the average immunohistochemistry staining scores of ADNP in 73 cases non-progression BC tissue and 55 cases progression BC tissue after chemotherapy. **D,** Kaplan-Meier method analyzed the no progression survival in 128 BC patients according to ADNP expression. * P <0.05.

We randomly collected the surgical fresh specimens from 43 BC patients, including 25 patients with tumor-progression and 18 patients with no-progression. The expression of ADNP mRNA was up-regulated in the patients who suffered tumor-progression versus in the patients with no-progression (*P*<0.05, Fig. 1A). Additionally, the immunohistochemistry of 128 BC tissues showed that ADNP protein was also significantly overexpressed in patients with tumor-progression (*P* <0.01, Table 1 and Fig. 1B, C). These data suggested that overexpression of ADNP associated with tumor-progression in patients treated with chemotherapy.

### Patients with high ADNP expression were associated with poor prognosis

In order to explore the relationship between ADNP expression and clinical parameters, we collected the clinical data of the 128 BC patients and scored the degree of ADNP antibody staining. The clinical data of these paraffin-embedded BC specimens showed in Table 1. Kaplan-Meier survival curve showed that the no-progression was significantly shorter in patients with high expression of ADNP versus in the patients with low ADNP expression (P <0.01, Fig. 1D). Cox regression analysis also showed that ADNP was a risk prognostic factor for bladder tumor-progression after chemotherapy (Table 2). In summary, these data supported that ADNP upregulation in BC tissues was associated with poor prognosis in patients treated with chemotherapy.

### ADNP increased the cisplatin-resistance in BC cells

**Figure 2:**
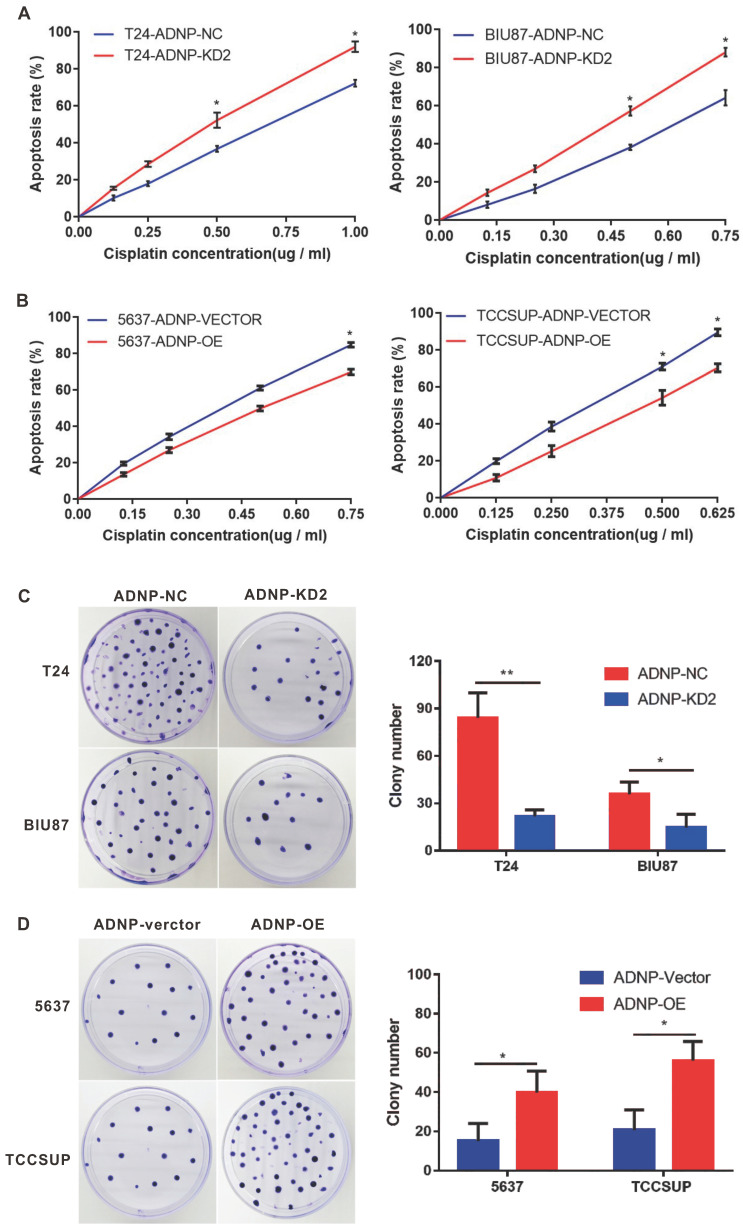
** ADNP increases the cisplatin-resistance in BC cells. A,** T24 and BIU87 cells were transfected with different ADNP-knockdown shRNA (KD-2) and negative control shRNA (NC) to establish the stable ADNP knockdown cell. CCK8 detected the apoptosis rate in BC cells under different concentrations of cisplatin. **B,** 5637 and TCCSUP cells transfected with ADNP overexpression plasmid (OE) and the empty vector ADNP (Vector) to establish the stable ADNP overexpressing cell. CCK8 detected the apoptosis rate of BC cells under different concentrations of cisplatin. **C,** Clone formation test to detect the growth of BC cells with the treatment of cisplatin between ADNP knockdown group and negative control group in T24 and BIU87. **D,** Clone formation test to detect the growth of BC cells treated with cisplatin between ADNP overexpressing group and blank control group in 5637 and TCCSUP. ** P <0.01, * P <0.05.

In previous studies, we used western blotting to detect the basic expression of ADNP protein in four BC cell lines, in which it was low in 5637 and TCCSUP. By contrast, ADNP expression was much high in T24 and BIU87. Therefore, we performed a series of functional experiments using constructed stable ADNP knockdown and overexpressed cells to test whether ADNP contributes to the sensitivities of cisplatin in BC cells. We knocked down ADNP in T24 and Biu87 cells, respectively. CCK8 test showed that Knocking down ADNP reduced cell viability in both types of cells, and the promoting effect became stronger with the increase of cisplatin concentration. (Fig. 2A). Similarly, ADNP knockdown in the cells under cisplatin treatment also significantly reduced the average number of colonies in the colony formation experiment (Fig. 2C). Furthermore, we overexpressed ADNP in 5637 and TCCSUP cell lines, both CCK8 and colony formation analysis showed that ADNP overexpression significantly increased the cell viability and average colony number (Fig. 2B and 2D). These results indicated that ADNP may induce the resistance of BC cells to cisplatin.

### ADNP accelerated migration and promoted EMT in BC cells

**Figure 3:**
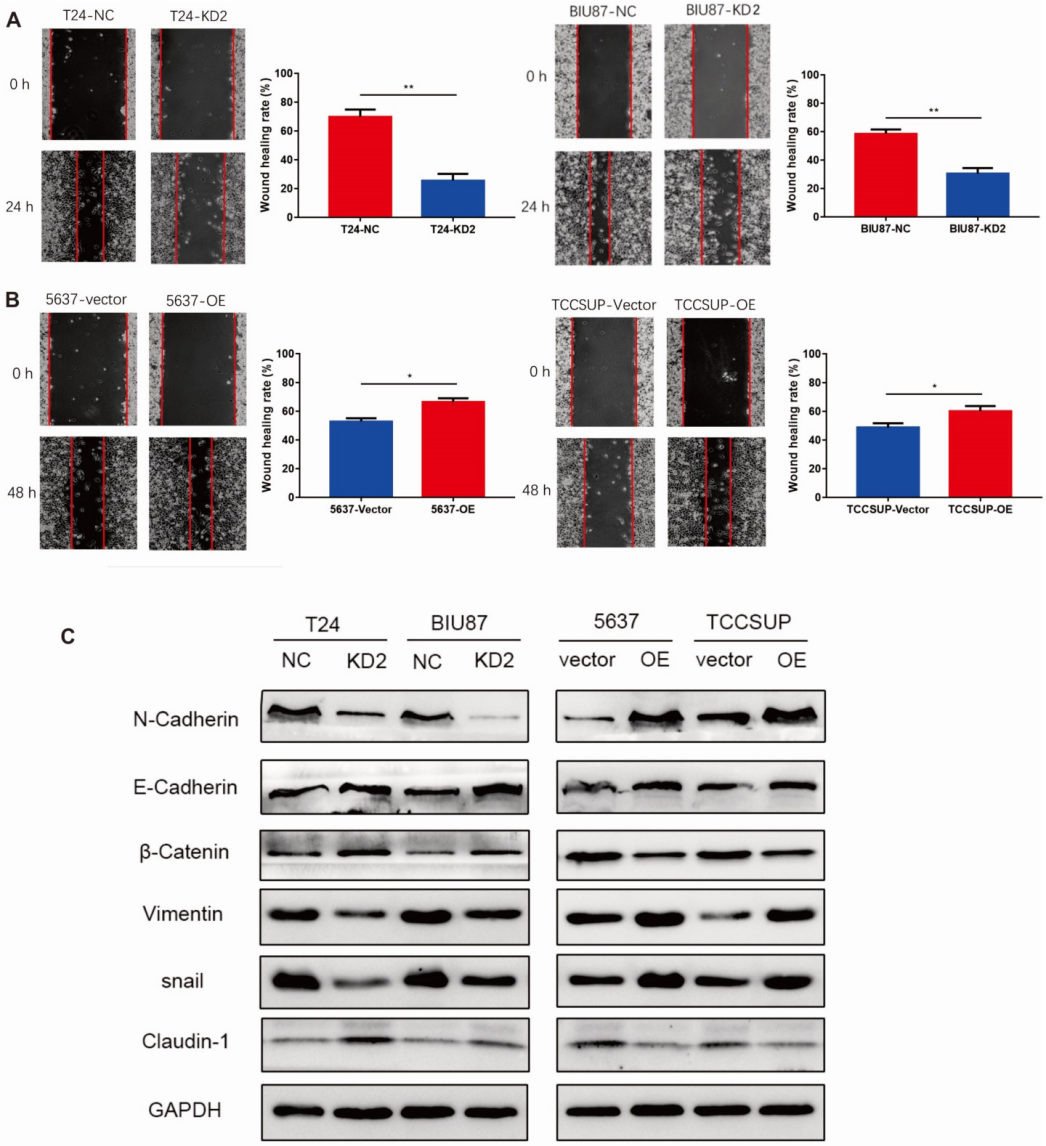
** ADNP accelerates migration of BC cells. A,** Wound healing assay was used for validating the effect of ADNP on migration in BC. **B,** Western blot was used to detect the expression of N-Cadherin, E-Cadherin, β-Catenin, Vimentin, snail, and Claudin-1 in the different groups. ** P <0.01, * P <0.05.

We performed wound healing experiment to explore the impact of ADNP on migration in BC cells. As shown in Fig. 3A, cells with ADNP knockdown exhibited delayed wound healing compared with NC group under cisplatin treatment. Overexpressed ADNP showed opposite results, suggesting that ADNP may promote the migration of BC cells under cisplatin treatment. Interestingly, compared with NC group under cisplatin treatment, ADNP-overexpression up-regulated the expression of mesenchymal cell markers including N-Cadherin, Vimentin and snail as detected by western blot analysis, while the expression of E-cadherin, β-Catenin and Claudin-1 were down-regulated. By contrast, cells with ADNP-knockdown showed the opposite (Fig. 3B). These data strongly suggested that ADNP induced EMT. Together, ADNP can increase the resistance of BC cells to cisplatin possibly by accelerating the migration and promoting EMT in BC cells.

### ADNP regulated the cisplatin-resistance of BC *in vivo*

**Figure 4:**
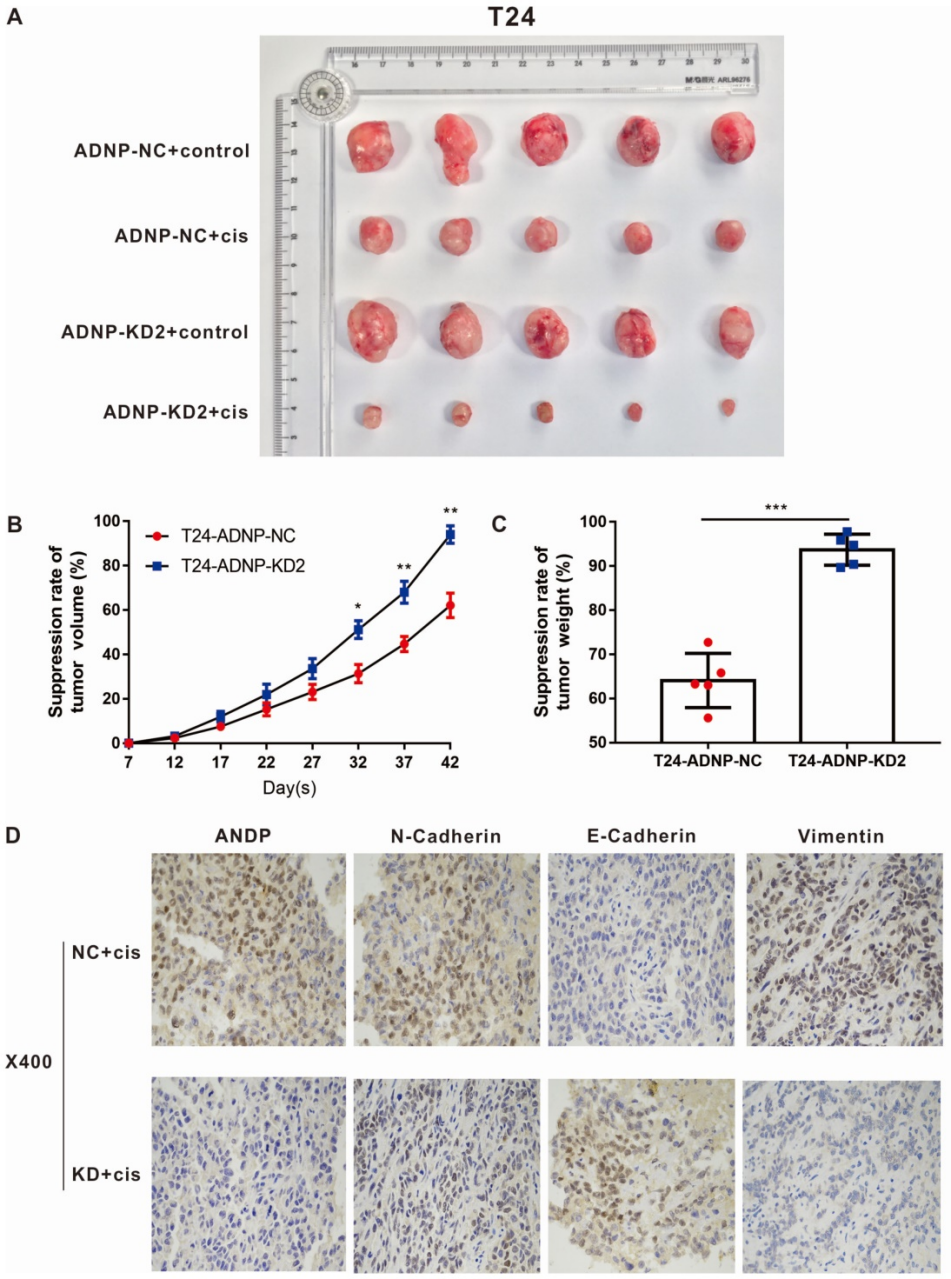
** ADNP regulates the cisplatin-resistance in BC *in vivo*. A,** 4 groups image of tumors dissected from NOD/SCID nude mice. **B,** Tumors volume was measured from the 7th day after tumor implantation, the continuous growth curve was drawn. **C,** Comparison of the average tumor resection weight of the control group and the knockdown group with the treatment of cisplatin. **D,** Immunohistochemical detection of representative images of ADNP, N-Cadherin, E-Cadherin, and Vimentin in two groups of nude mice. *** P <0.001, ** P <0.01, * P <0.05.

In order to verify the experimental results *in vitro*, we constructed xenograft bladder tumor models using T24 bladder cells in NOD/SCID nude mice to evaluate the effect of ADNP on cisplatin response *in vivo*. As shown in Fig. 4A-C, cisplatin were associated with lower ability of tumor forming. ADNP knockdown inhibited tumor formation and higher drug response rate. Using immunohistochemistry on isolated nude mouse tumor tissues, the results confirmed that ADNP knockdown significantly down-regulated the expression of ADNP, N-Cadherin, and Vimentin after cisplatin intervention, while E-Cadherin expression was the opposite (Fig. 4D). These results *in vivo* suggested that ADNP may play an important role in the development of cisplatin resistance in BC.

### ADNP activated TGF-β/Smad signaling pathway

**Figure 5:**
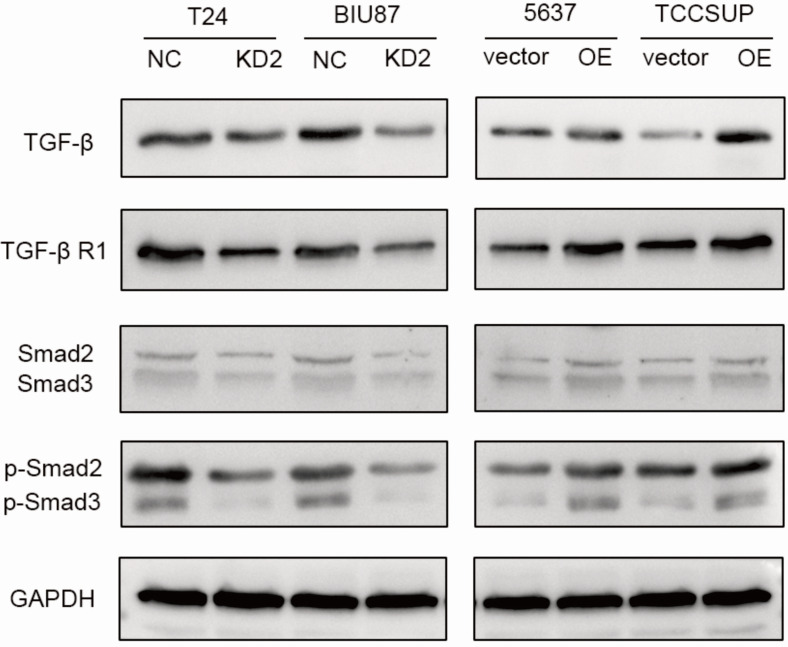
** ADNP activates TGF-β pathway in BC cells.** BC cells were treated with cisplatin (0.125 µg/ml, 48h). The expression of the key proteins in the TGF-β/Smad, including TGF-β, TGF-βR1, Smad2/Smad3, p-Smad2/p-Smad3 were detected in BC cells by using western blot.

To further study the possible molecular mechanism why ADNP regulated EMT and cisplatin-resistance, we treated the BC cells with cisplatin (0.125 ug/ml, 48 h) and used western bolt to explore the protein expression of the key proteins in the TGF-β/Smad, which is classic signaling pathway in EMT processes. As shown in the experiments with ADNP knockdown in T24 and BIU87 BC cells, expression of TGF-β, TGF-βR1, Smad2, Smad3, p-Smad2 and p-Smad3 was significantly decreased, while overexpression of ADNP in 5637 and TCCSUP BC cells produced the opposite effects (Fig. 5). These results suggested that ADNP activated the TGF-β/Smad signaling pathway. ADNP promoted EMT and induced cisplatin resistance in BC cells, which may be related to TGF-β/Smad signaling pathway.

## Discussion

In this study, we analyzed the relationship between ADNP and cisplatin-resistance in human BC. Our results, for the first time, indicate that ADNP expression was higher in progression BC compared to non-progression BC. Patients with high ADNP expression in BC had significantly shorter survival after chemotherapy. Here we provided the potential evidences that ADNP induced cisplatin-resistance.

ADNP encodes a protein that is involved in the development of the nervous system in embryos 33. Besides, ADNP gene is also closely linked with tumor cells proliferation, invasion as well as migration 15. Wound healing experiments showed that ADNP promoted cell migration 15, 34. A recent study showed that the expression of ADNP enhanced the adaptive ability of malignant peripheral nerve sheath tumors to survive in a malignant environment 16, 35, 16. The expression of ADNP in bladder was related to age and sex, and is higher in young men 36. Here we found that the high expression of ADNP leaded to unfavorable prognosis in patients treated with chemotherapy. Thus, we hypothesized that ADNP may promote cells proliferation and cell migration, thereby increasing the chemoresistance in BC. In this study, we verified our hypotheses by using *in vitro* experiments. Our results revealed that ADNP knockdown in BC cell lines significantly reduced cells proliferation and cell migration under cisplatin, while ADNP upregulation showed the opposite results. These results supported that ADNP had an effect on BC chemoresistance.

EMT is considered to be closely related to tumor progression and chemotherapy resistance 10. Gene expression alterations during EMT processes resulted in many phenotypic changes such as changes in cell morphology, loss of adhesion, and acquisition of stem cell-like features 37. In most cases, the loss of E-cadherin is a hallmark of EMT. For instance, MCF-7 breast cancer cell line resistant to adriamycin and the ZR-75-B breast cancer cell line resistant to vincristine exhibited EMT and vimentin high expression, while E-cadherin, formation of desmosomes and tight junction expression were reduced 38. In this study, we used immunoblotting to analyze the expression of EMT-related biomarkers in BC cell lines. ADNP upregulation increased the expression of mesenchymal biomarkers like N-cadherin, vimentin as well as snail, whereas it reduced the expression of epithelial proteins like E-cadherin, β-catenin and Claudin-1, which consist with previous founding. By contrast, ADNP downregulation showed the opposite results, indicating that ADNP promoted the EMT in BC cells. A close relationship between ADNP-induced EMT and cisplatin-resistance were also established on mice models. Subcutaneous xenotransplantation was used to establish a model in nude mice. The results showed that cisplatin were associated with lower ability of tumor forming. ADNP knockdown inhibited tumor formation and higher drug response rate. ADNP knockdown significantly down-regulated the expression of ADNP, N-Cadherin, and Vimentin after cisplatin intervention, while E-Cadherin expression was the opposite. These data were consistent the research on lung cancer. The growth rate of primary tumor in mice was reduced by 60% by treatment with the cyclophosphamide (a chemotherapeutic drug). However, as compared with epithelial-type cancer cells, EMT-positive cells in primary tumors were resistant to drug induced cell apoptosis. The number of cells were without significantly decreasing under chemotherapy 39. Above data revealed that EMT might have contribution on drug resistance in BC.

Transforming growth factor beta (TGF-β), which played an important role in cell proliferation, differentiation, embryogenesis and morphogenesis, is thought to be a main pathway of the EMT process in cancer 40. In classic TGF-β/Smad signaling, TGF-β ligand binded to the type II TGF-β receptor (TβRII) on the cell membrane to form the TGF-β/TβRII complex to activate the type I TGF-β receptor (TβRI) 41, 42. Phosphorylation of TβRI activated phosphorylation of Smad2 and Smad3 in cytoplasm 43. Phosphorylation of Smad2/3 caused a trimer formation between phosphorylated Smad2/3 and Smad 4 44. The receptor activated Smad complex activated or inhibited its target gene promoter, thereby promoting EMT 45. Studies showed that long-term exposure to EGFR-TKIs activated TGF-β/Smad signaling and promoted the EMT phenotype in non-small cell lung cancer 9. In line with this, we knocked down ADNP in T24 and BIU87 BC cells and found the expression of TGFβ, TGF-βRI and p-Smad2/3 were decreased, in contrast, overexpression of ADNP in 5637 and TCCSUP BC cells showed opposite results, indicating that ADNP promoted cell migration and EMT, thereby inducing cisplatin resistance, which may be related to TGF-β/Smad signaling pathway. TGF-β belongs to the TGF-β superfamily, which regulates cell growth and differentiation, and can transform the phenotype of normal fibroblasts. The TGF-β pathway was affected by changes in epithelial cell adhesion molecules 46. Previous studies have shown that cell growth and migration were regulated by cell adhesion (such as cadherins, selectins), while ADNP promoted cell adhesion 47. Therefore, we speculated that ADNP may affect the activation of TGF-β signaling pathway by regulating cell adhesion, thereby promoting EMT and increasing the resistance of BC cells to cisplatin, which is the content of our next study.

In summary, our study showed that ADNP was significantly upregulated and was closely related to unfavorable prognosis in progression BC after chemotherapy. ADNP promoted cell migration and EMT, thereby inducing cisplatin resistance, which may be related to TGF-β/Smad signaling pathway. Our results provided a mechanistic explanation for the involvement of ADNP in BC progression under chemotherapy. ADNP may be a novel molecular target for predicting prognosis and cancer therapy in patients with BC after chemotherapy.

## Figures and Tables

**Table 1:** Correlations of ADNP expression and clinicopathological features of bladder cancer patients

Characteristics	n	ADNP expression		*P*-value
		Low (n=72)	High (n=56)	
**Age (y)**				
≥60	72 (56.3%)	44	28	0.209
<60	56 (43.8%)	28	28	
**Gender**				
Male	61 (47.7%)	39	22	0.094
Female	67 (52.3%)	33	34	
**T stage**				
T2	85 (66.4%)	56	29	0.002
T3, T4	43 (33.6%)	16	27	
**Tumor multiplicity**				
Unifocal	81 (63.3%)	52	29	0.017
Multifocal	47 (36.7%)	20	27	
**Tumor size (cm)**				
<3	76 (59.4%)	34	42	0.002
≥3	52 (40.6%)	38	14	
**Vital states**				
non-progression	73 (57.0%)	52	21	<0.001
progression	55 (43.0%)	20	35	

**Table 2:** Univariate and multivariate Cox-regression analyses of prognostic factors in bladder cancer patients

Variables	HR	Univariate analysis		HR	Multivariate analysis	
		95% CI	*p*-value		95% CI	*p*-value
Age	1.129	0.655-1.129	0.655	-	-	-
Sex	1.225	0.719-2.088	0.455	-	-	-
T stage	3.823	2.232-6.549	<0.001	2.513	1.398-4.517	0.002
Tumor multiplicity	2.839	1.663-4.848	<0.001	1.923	1.073-3.447	0.028
Tumor size	0.360	0.196-0.662	<0.001	0.484	0.256-0.917	0.026
ADNP	3.362	1.936-5.838	<0.001	2.091	1.130-3.867	0.019

Abbreviations: HR: hazard ratio; CI: confidence interval.

## References

[1] Bhanvadia S K (2018). Bladder Cancer Survivorship. Curr Urol Rep.

[2] Chen J, Li Y, Li Z (2020). LncRNA MST1P2/miR-133b axis affects the chemoresistance of bladder cancer to cisplatin-based therapy via Sirt1/p53 signaling. J Biochem Mol Toxicol.

[3] Kitamura T, Suzuki M, Nishimatsu H (2010). Final report on low-dose estramustine phosphate (EMP) monotherapy and very low-dose EMP therapy combined with LH-RH agonist for previously untreated advanced prostate cancer. Aktuelle Urol.

[4] Li Y, Li G, Guo X (2020). Non-coding RNA in bladder cancer. Cancer Lett.

[5] Jiménez-Garduño A M, Mendoza-Rodríguez M G, Urrutia-Cabrera D (2017). IL-1beta induced methylation of the estrogen receptor ERalpha gene correlates with EMT and chemoresistance in breast cancer cells. Biochem Biophys Res Commun.

[6] Li H, Li J, Chen L (2019). HERC3-Mediated SMAD7 Ubiquitination Degradation Promotes Autophagy-Induced EMT and Chemoresistance in Glioblastoma. Clin Cancer Res.

[7] Yang Y, Yao J H, Du Q Y (2019). Connexin 32 downregulation is critical for chemoresistance in oxaliplatin-resistant HCC cells associated with EMT. Cancer Manag Res.

[8] Zeng D, Liang Y K, Xiao Y S (2020). Inhibition of Notch1 reverses EMT and chemoresistance to cisplatin via direct downregulation of MCAM in triple-negative breast cancer cells. Int J Cancer.

[9] Soucheray M, Capelletti M, Pulido I (2015). Intratumoral Heterogeneity in EGFR-Mutant NSCLC Results in Divergent Resistance Mechanisms in Response to EGFR Tyrosine Kinase Inhibition. Cancer Res.

[10] Kotiyal S, Bhattacharya S (2014). Breast cancer stem cells, EMT and therapeutic targets. Biochem Biophys Res Commun.

[11] Hashmi F, Liu M, Shen S (2016). Phospholipase C gamma mediates endogenous brain-derived neurotrophic factor - regulated calcitonin gene-related peptide expression in colitis - induced visceral pain. Mol Pain.

[12] Karagoz K, Mehta G A, Khella C A (2019). Integrative proteogenomic analyses of human tumours identifies ADNP as a novel oncogenic mediator of cell cycle progression in high-grade serous ovarian cancer with poor prognosis. EBioMedicine.

[13] Zamostiano R, Pinhasov A, Gelber E (2001). Cloning and characterization of the human activity-dependent neuroprotective protein. J Biol Chem.

[14] Gozes I, Yeheskel A, Pasmanik-Chor M (2015). Activity-dependent neuroprotective protein (ADNP): a case study for highly conserved chordata-specific genes shaping the brain and mutated in cancer. J Alzheimers Dis.

[15] Blaj C, Bringmann A, Schmidt E M (2017). ADNP Is a Therapeutically Inducible Repressor of WNT Signaling in Colorectal Cancer. Clin Cancer Res.

[16] Castorina A, Giunta S, Scuderi S (2012). Involvement of PACAP/ADNP signaling in the resistance to cell death in malignant peripheral nerve sheath tumor (MPNST) cells. J Mol Neurosci.

[17] Zhu S, Xu Z, Zeng Y (2020). ADNP Upregulation Promotes Bladder Cancer Cell Proliferation via the AKT Pathway. Front Oncol.

[18] Gupta S, Chaudhary S, Bubber P (2019). Epidemiology and genetic diversity of group A rotavirus in acute diarrhea patients in pre-vaccination era in Himachal Pradesh, India. Vaccine.

[19] Shu-Jun X, Kang W, Min-Hong Z (2017). Comparison of three different methods for isolating RNA from Oncomelania hupensis. Zhongguo Xue Xi Chong Bing Fang Zhi Za Zhi.

[20] Wang S Q, Xu B, Liu J (2014). Effect of RNA interference targeting Schistosoma japonicum aldose reductase gene. Zhongguo Ji Sheng Chong Xue Yu Ji Sheng Chong Bing Za Zhi.

[21] Li T S C, Yawata T, Honke K (2014). Efficient siRNA delivery and tumor accumulation mediated by ionically cross-linked folic acid-poly(ethylene glycol)-chitosan oligosaccharide lactate nanoparticles: for the potential targeted ovarian cancer gene therapy. Eur J Pharm Sci.

[22] Kang J F, Hu H H, Chen R (2008). *In vitro* observation on the apoptosis induced by H2O2 in protoscolex of Echinococcus granulosus]. Zhongguo Ji Sheng Chong Xue Yu Ji Sheng Chong Bing Za Zhi.

[23] Nguyen H M, Sako K, Matsui A (2017). Ethanol Enhances High-Salinity Stress Tolerance by Detoxifying Reactive Oxygen Species in Arabidopsis thaliana and Rice. Front Plant Sci.

[24] Wang L, Ouyang F, Liu X (2016). Overexpressed CISD2 has prognostic value in human gastric cancer and promotes gastric cancer cell proliferation and tumorigenesis via AKT signaling pathway. Oncotarget.

[25] Chen Y, Jiang H, Cao J (2015). Influence of Photodynamic Therapy on Apoptosis and Invasion of Human Cholangiocarcinoma QBC939 Cell Line. Chin Med Sci J.

[26] Gong S, Tao Z, Liu X (2014). An underlying prognosis predictor of hepatocellular carcinoma: Oncoprotein 18. Biomed Rep.

[27] Wilson C, Lukowicz R, Merchant S (2017). Quantitative and Qualitative Assessment Methods for Biofilm Growth: A Mini-review. Res Rev J Eng Technol.

[28] Guzman C, Bagga M, Kaur A (2014). ColonyArea: an ImageJ plugin to automatically quantify colony formation in clonogenic assays. PLoS One.

[29] Arranz-Valsero I, Soriano-Romaní L, García-Posadas L (2014). IL-6 as a corneal wound healing mediator in an *in vitro* scratch assay. Exp Eye Res.

[30] Perfetto B, Stellavato A, Melito A (2012). A time-lapse approach to examine chromium and nickel effects on wound healing *in vitro*. J Immunotoxicol.

[31] Wang B, Chen Q, Cao Y (2016). LGR5 Is a Gastric Cancer Stem Cell Marker Associated with Stemness and the EMT Signature Genes NANOG, NANOGP8, PRRX1, TWIST1, and BMI1. PLoS One.

[32] Lehmann C, Friess T, Birzele F (2016). Superior anti-tumor activity of the MDM2 antagonist idasanutlin and the Bcl-2 inhibitor venetoclax in p53 wild-type acute myeloid leukemia models. J Hematol Oncol.

[33] Furman S, Hill J M, Vulih I (2005). Sexual dimorphism of activity-dependent neuroprotective protein in the mouse arcuate nucleus. Neurosci Lett.

[34] Mollinedo P, Kapitansky O, Gonzalez-Lamuño D (2019). Cellular and animal models of skin alterations in the autism-related ADNP syndrome. Sci Rep.

[35] Suzuki M, Liu M, Kurosaki T (2011). Association of rs6983561 polymorphism at 8q24 with prostate cancer mortality in a Japanese population. Clin Genitourin Cancer.

[36] Kapitansky O, Sragovich S, Jaljuli I (2020). Age and Sex-Dependent ADNP Regulation of Muscle Gene Expression Is Correlated with Motor Behavior: Possible Feedback Mechanism with PACAP. Int J Mol Sci.

[37] Chen T, You Y, Jiang H (2017). Epithelial-mesenchymal transition (EMT): A biological process in the development, stem cell differentiation, and tumorigenesis. J Cell Physiol.

[38] Sommers C L, Heckford S E, Skerker J M (1992). Loss of epithelial markers and acquisition of vimentin expression in adriamycin- and vinblastine-resistant human breast cancer cell lines. Cancer Res.

[39] Kuczynski E A, Krueger J, Chow A (2018). Impact of Chemical-Induced Mutational Load Increase on Immune Checkpoint Therapy in Poorly Responsive Murine Tumors. Mol Cancer Ther.

[40] David C J, Huang Y H, Chen M (2016). TGF-β Tumor Suppression through a Lethal EMT. Cell.

[41] Zhang J, Wang Y, Weng H (2019). Management of non-muscle-invasive bladder cancer: quality of clinical practice guidelines and variations in recommendations. BMC Cancer.

[42] Deng G, Chen L, Zhang Y (2018). Fucosyltransferase 2 induced epithelial-mesenchymal transition via TGF-beta/Smad signaling pathway in lung adenocarcinaoma. Exp Cell Res.

[43] Chen H, Cai J, Wang J (2020). Targeting Nestin(+) hepatic stellate cells ameliorates liver fibrosis by facilitating TβRI degradation. J Hepatol.

[44] Martin-Malpartida P, Batet M, Kaczmarska Z (2017). Structural basis for genome wide recognition of 5-bp GC motifs by SMAD transcription factors. Nat Commun.

[45] Sakai S, Ohhata T, Kitagawa K (2019). Long Noncoding RNA ELIT-1 Acts as a Smad3 Cofactor to Facilitate TGFβ/Smad Signaling and Promote Epithelial-Mesenchymal Transition. Cancer Res.

[46] Martin M E, Reaves D K, Jeffcoat B (2019). Silver nanoparticles alter epithelial basement membrane integrity, cell adhesion molecule expression, and TGF-β1 secretion. Nanomedicine.

[47] Greenberg D A (2003). Linking acquired neurodevelopmental disorders to defects in cell adhesion. Proc Natl Acad Sci U S A.

